# Preservation of the Subtalar Joint Determines Outcomes in a 10-Year Evaluation of Ankle Arthrodesis

**DOI:** 10.3390/jcm12093123

**Published:** 2023-04-25

**Authors:** Rebecca Sell, Magalie Meinert, Eva Herrmann, Yves Gramlich, Alexander Klug, Oliver Neun, Reinhard Hoffmann, Sebastian Fischer

**Affiliations:** 1 Department of Foot and Ankle Surgery, Berufsgenossenschaftliche Unfallklinik Frankfurt am Main, 60389 Frankfurt am Main, Germany; 2 Department for Trauma and Orthopaedic Surgery, Berufsgenossenschaftliche Unfallklinik Frankfurt am Main, 60389 Frankfurt am Main, Germany; 3 Institut für Biostatistik und Mathematische Modellierung, Goethe-Universität Frankfurt am Main, Theodor-Stern-Kai 7, 60596 Frankfurt am Main, Germany

**Keywords:** ankle arthrodesis, tibiotalocalcaneal arthrodesis, subtalar arthrodesis, gait pattern, posttraumatic osteoarthritis

## Abstract

Posttraumatic osteoarthritis may lead to surgical fusion of the ankle joint if non-surgical therapy fails. The indication for a fusion of the joint is based on the pain and disability of the patient, radiographic imaging, and surgeon experience, with no strict guidelines. We aimed to compare outcomes after tibiotalocalcaneal arthrodesis (TTCA) and tibiotalar arthrodesis (TTA) to highlight the functional importance of the subtalar joint. In total, 432 patients with ankle arthrodesis were retrospectively enrolled. Group A (*n* = 216) underwent TTCA; group B (*n* = 216) underwent TTA. Demographics, Olerud & Molander Ankle Score (OMAS), Foot Function Index (FFI-D), and Short Form-12 Questionnaire (SF-12) were recorded at a mean follow-up of 6.2 years. The mean OMAS was 50.7; the mean FFI-D was 68.9; the mean SF-12 physical component summary was 39.1. These scores differed significantly between the groups (*p* < 0.001). The overall revision rate was 18%, primarily for revision of non-union and infection (*p* < 0.001). Approximately 16% of group A and 26% of group B were able to return to previous work (*p* < 0.001). Based on significantly worse clinical scores of TTCA compared to TTA and the prolonged downtime and permanent incapacity, the indication for a generous subtalar joint arthrodesis with planned ankle arthrodesis should always be critically examined.

## 1. Introduction

Rare primary arthroses of the ankle joint account for less than ten percent of all cases. Additionally, traffic accidents and sports injuries lead to serious fractures of the ankle joint with posttraumatic osteoarthritis [1]. However, chronic instabilities due to insufficiency of the inner and outer ligaments of the upper ankle joint, as well as acute and chronic syndesmosis injuries with resultant chronic instability, are also possible causes, and habitual malpositions with an axial deviation of the hindfoot or entire leg axis also favor such signs of wear and tear [2]. If non-surgical therapy, such as adjustment of footwear, nonsteroidal anti-inflammatory drugs, and physiotherapy, fails, and joint preservation is no longer possible, the indication for arthrodesis of the ankle joint arises.

In this context, it is known that a subsequent conversion of a total ankle replacement into a tibiotalar arthrodesis is inferior to a primary fusion [3,4]. The idea that previously mild osteoarthritis of the subtalar joint may develop into severe osteoarthritis leads to a discussion of early indications for tibiotalocalcaneal arthrodesis (TTCA).

Regardless of the radiological findings, complaints of pain surrounding the subtalar joint cannot always be reliably differentiated when tibiotalar arthrodesis (TTA) is indicated. The indication for TTA or TTCA then depends primarily on the experience of the treating surgeon, in addition to the patient’s disability and pain.

The outcomes of TTA and TTCA are sometimes unsatisfactory and are associated with low score values due to the associated restriction of movement and the long duration of pain and suffering [5,6]. We aimed to compare the clinical outcomes of TTCA and TTA in a direct comparison using a demographically comparable and large patient population.

## 2. Materials and Methods

### 2.1. Population

**Figure 1:**
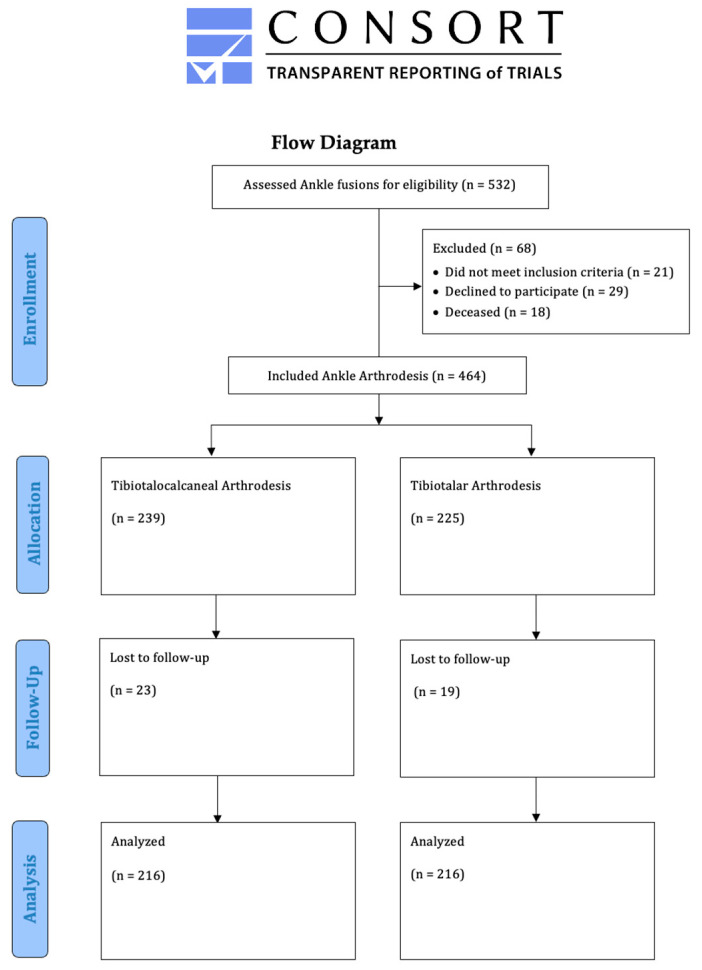
Study flow chart.

Between 2010 and 2022, 432 patients with ankle arthrodesis (278 males and 154 females, mean age: 64 years [range: 27–93 years]) were retrospectively enrolled in this comparative monocentric study. Group A (*n* = 216) underwent TTCA; group B (*n* = 216) underwent TTA. Both groups were equally distributed in terms of demographics (Table 1). All patients were seen during foot surgery consultations at the study center (Figure 1). The diagnosis of end-stage posttraumatic osteoarthritis of the ankle was made on the basis of clinical examination, obligatory weight-bearing radiographs, and computer tomography.

All patients underwent TTCA or TTA at the study center by five surgeons with the same expertise in this type of surgery. The mean follow-up duration for clinical outcomes was 6.2 years (range: 12–154 months). All procedures were performed in accordance with the 1964 Helsinki Declaration and its later amendments. The ethics committee of the institutional review board approved this study (2022-2883-evBO).

### 2.2. Inclusion and Exclusion Criteria

Patients older than 18 years were included; there was no maximum age limit. Written informed consent was required prior to participation. The indication for surgery was based on underlying painful end-stage posttraumatic osteoarthritis of the tibiotalar and additional subtalar joint leading to TTCA or TTA. Only surgeries performed at the study center were included. Destruction of the ankle joint due to rheumatic disease or malignant neoplasm of bone, such as osteosarcoma with multiple reconstructions and eventual tibiotalar fusion, were not included.

### 2.3. Surgical Procedures

The decision for TTCA or TTA on the basis of the objectifiable radiological criteria was largely guided by the surgeon’s personal experience, the expected osteoarthritis of the subtalar joint, and the patient’s expectations. Uniform criteria could only be completely delimited at follow-up. The surgical procedures were performed under general anesthesia or, less frequently, under spinal anesthesia, and a tourniquet was obligatorily applied to the thigh. The patient was placed in a supine position for both procedures.

**Figure 2:**
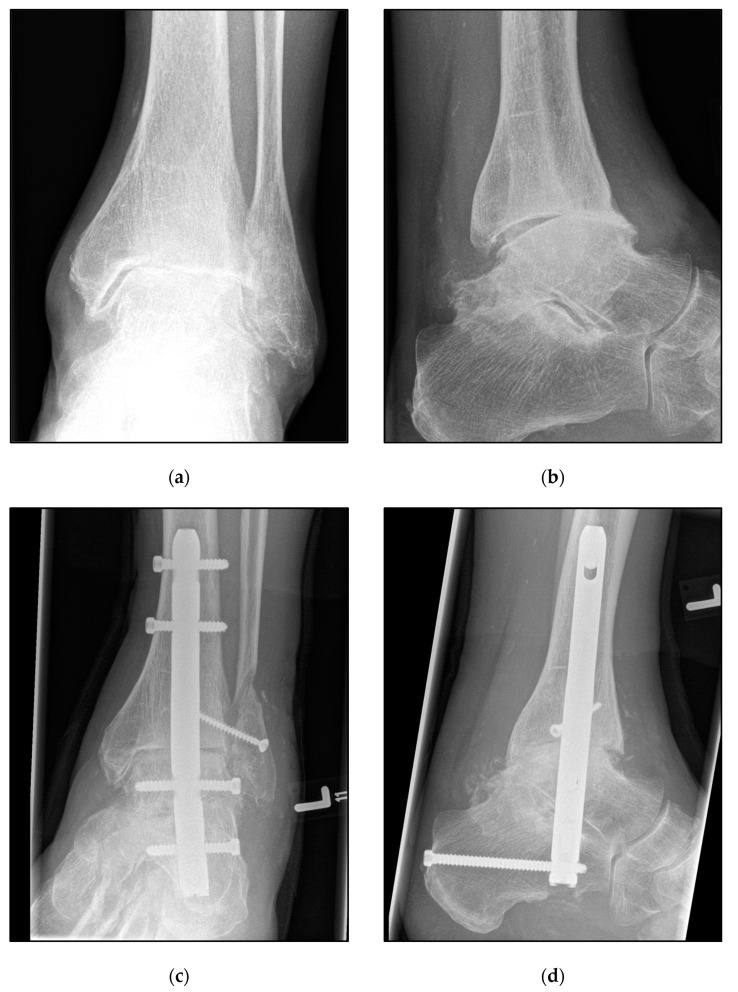
Pre- and postoperative radiographic findings of end-stage posttraumatic arthritis of the left ankle of a 79-year-old male treated with a tibiotalocalcaneal arthrodesis (TTCA) T2™ Ankle Arthrodesis Nail, 150 × 12 mm. (**a**,**b**) Anteroposterior view, preoperative. (**c**,**d**) Anteroposterior view, 3 months postoperative.

For TTCA, the approach was usually along the lateral malleolus, which is osteotomized and decorticated 5–10 cm proximal to the tip of the malleolus, depending on the size of the patient and pre-ordered destruction of the ankle joint. The tibiotalar and subtalar joint was then dissected via this approach, with the removal of any remaining cartilage and resection of the destroyed subchondral sclerosis. All TTCAs were performed by implantation of a hindfoot fusion nail with 5° valgus. The diameter and length of the nail were chosen to be between 150 mm and 300 mm according to preoperative planning and intraoperative findings (Figure 2). A shorter nail with a diameter of 12 mm was the most common. Interposition autologous or allogenic cancellous bone grafting was performed in less than 20% of cases.

**Figure 3:**
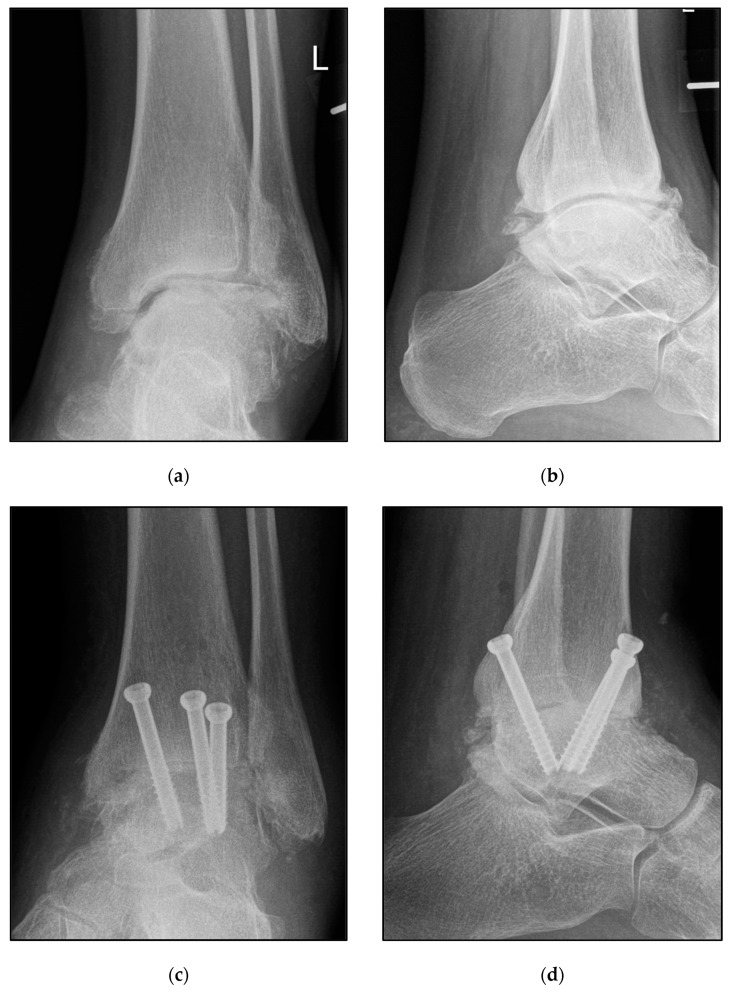
Pre- and postoperative radiographic findings of end-stage posttraumatic arthritis of the left ankle of a 44-year-old male treated with tibiotalar arthrodesis (TTA) using cannulated screws (diameter 6.5 mm). (**a**,**b**) Anteroposterior view, preoperative. (**c**,**d**) Anteroposterior view, 8 weeks postoperative.

TTA was regularly performed via an anterior approach between the tibialis anterior and the extenso hallucis longus tendon. After the prescribed preparation of the joint, a fusion was performed by inserting 2–3 converging cannulated screws (diameter of 6.5 or 8 mm) or an anterior fusion plate (Figure 3). Other approaches, such as lateral, posterolateral, and medial, as well as combined approaches, were also used where necessary. Nevertheless, treatment via the anterior approach was the most common, at over 80%. Regardless of the technical implementation, both the TTCA and the TTA were designed to be neutral in both the coronal and the sagittal plane, with a physiological valgus of the hindfoot of 5°.

### 2.4. Rehabilitation Protocol

The protocol following TTCA and TTA was the same. The post-treatment scheme involved wearing an orthotic boot (e.g., VACOped™) for a total of 12 weeks and ambulating on the forearm or armpit crutches. For the first 6 weeks, patients were required to wear the boot for 24 h a day with merely sole contact; removal of the boot for personal hygiene and physiotherapy was permitted.

After an X-ray examination, the boot was worn for an additional 6 weeks, with a gradual increase in load. During this time, the boot could be removed at night. At 12 weeks after surgery, computed tomography was carried out, and the footwear was orthopedically adapted for everyday use.

### 2.5. Assessment Methods

Demographic data, including age, body mass index (BMI), pre-existing conditions (such as those associated with syndrome-x), and nicotine abuse, were obtained for each patient. The Olerud & Molander Ankle Score (OMAS), Foot Function Index in its validated German version (FFI-D), the Short Form-12 Questionnaire (SF-12), and the type and number of revisions were recorded as part of the follow-up (Table 1).

### 2.6. Statistical Analysis

The primary goal was to compare significant differences in the outcome of TTCA and TTA using a representative number of patients to illustrate the power of the included data with a mean follow-up time of 6.2 years. Due to the retrospective design, there is no case number calculation. So far, monocentric studies with comparable questions have presented significantly smaller populations [7,8]. All statistical analyses were performed using SPSS v. 23 software (IBM Dtl. GmbH, Ehningen, Germany). Furthermore, descriptive and explorative statistical analyses for the queried scores, including within-group means, medians, minima and maxima, and standard deviations, were applied. Student’s *t*-test and ANOVA were used. The power of the study was 0.8, and the significance level was set to *p* < 0.05, with a 95% confidence interval.

## 3. Results

After an average postoperative follow-up of 74 months (range: 12–154 months), the mean OMAS and FFI-D scores were 50.7 (TTCA: 43.0; TTA: 58.2) and 68.9 points, respectively. The difference was significant, as was the physical component summary of the SF-12 (mean: 39.1; TTCA: 33.5; TTA: 42.5), (*p* < 0.001).

The ability of the patient to return to their job also differed significantly: in the TTCA group, around 15% returned successfully; in the TTA group, 26% (*p* < 0.001). Only the mental component summary of the SF-12 showed no significant difference, with a mean value of 50.6 for all patients (*p* = 0.369). In a free survey, one-third of patients reported that their gait was as expected after the arthrodesis, one-third reported that it was worse, and one-third managed better than expected before surgery. The distribution applied equally to both groups.

### Complications

The overall revision rate was approximately 19%, with a significantly higher proportion in the TTCA group (all *n* = 80; TTCA: *n* = 64 (29.6%), TTA: *n* = 16 (7.41%); *p* < 0.001). Most revisions had to be performed due to non-union and infections. In addition, minor complications, such as delayed wound healing, swelling, discomfort, and cramps, were recorded (Table 2).

## 4. Discussion

TTCA and TTA for the treatment of end-stage posttraumatic osteoarthritis of the ankle yielded significant differences in our validated scores. If an additional subtalar arthrodesis is necessary, in addition to the pure tibiotalar arthrodesis, this represents a massive impairment of quality of life. These patients will elicit significantly poorer results compared to those undergoing isolated tibiotalar arthrodesis.

The aim of TTCA and TTA is the relief of pain caused by end-stage posttraumatic arthrosis, as well as to straighten possible malpositions and establish stability. Both methods are established in this regard. However, there is still no clear guideline as to which patients benefit from subtalar arthrodesis in the context of ankle fusion. Direct comparisons, especially in studies with a population with only terminal posttraumatic osteoarthritis of the ankle that is comparable in terms of risk profile and demographic data, only show the results of a small number of cases [7]. The results of TTCA and TTA vary from good for bone consolidation, reduced postoperative complications, and improvement of pain and quality of life, to the suggestion that additionally fusing the subtalar joint does not cause greater movement restriction [8,9,10]. Both these statements are difficult to understand on the basis of our data. Rather, we have confirmed that the subtalar joint plays a decisive role in mobility, especially in the case of an already fused tibiotalar joint. The additional requirement of a fusion of the subtalar joint leads to a considerable restriction of mobility with a corresponding reduction in quality of life. Regardless of this assessment, the results of the quality of life scores for TTCA and TTA reflect the respective results of the current literature without significant deviations [11,12,13].

Since no relevant differences could be determined from the subgroup analysis of the respective arthrodesis procedures in the TTA group, a separate presentation of the results was not carried out. This is in accordance with Prissel et al., who also showed no significant difference in clinical and radiological outcomes with similar complication rates after ankle arthrodesis using anterior locking plate fixation or converging screws [14]. Thus, the present data confirm the biomechanical and clinical studies that put the importance of the screw diameter and number into perspective [15]. In our procedures, two, three, or even four converging cannulated screws were inserted in the TTA group. An additional arthrodesis of the distal tibiofibular joint as part of the TTCA or TTA also showed no influence on the results and was carried out in about half of all cases. The clinical impression that simultaneous arthrodesis of the distal tibiofibular joint has no influence on the fusion rate of the tibiotalar joint and the further outcome was confirmed by Schlickewei et al. [16].

The interposition of cancellous bone also showed no influence on the data presented [17,18]. As a rule, there is no need for autologous cancellous bone grafting in the context of ankle arthrodesis, as confirmed by systematic reviews. As in the present case, the underlying studies lack a prospective comparison and, in the case of autologous cancellous bone grafting, an objectifiable representation of the previous bony defect size [18].

We also found that the mental component summary of SF-12 was the only parameter that did not show a significant difference (*p* = 0.369). An obvious explanation for this may be that patients in whom an additional fusion of the subtalar joint was necessary were assumed to have an even worse function. In addition, these patients presumably come from a worse initial function, but this cannot be evaluated on the basis of the available data. Either way, the results of the mental component summary of SF-12 of the present study deviate from those of the current literature in patients after TTCA, TTA, and even after total ankle replacement [10,19,20,21].

Even though the stated complication rates of 19% for TTCA and TTC remain high, these results differ considerably from those in the literature of up to a frightening 50% [22,23,24]. The complication rate for TTCA is approximately four times higher than that for TTA (30% vs. 7%, respectively). TTCA should, therefore, be considered with appropriate restraint. One conceivable explanation would be the higher proportion of smokers in the TTCA group. In addition, the procedure immanent greater soft tissue damage.

This study had some limitations. First, this was a monocentric study with a retrospective design and clinical scores and the extent of posttraumatic damage to the tibiotalar and subtalar joints was not collected preoperatively. This makes it particularly difficult to assess the choice of implant and the need for cancellous bone grafting. Second, the indication for TTCA and TTA was presumably influenced by surgeon experience.

## 5. Conclusions

Based on the present data of significantly worse clinical score results of TTCA compared to TTA, the fourfold complication rate, and the prolonged downtime and possible permanent incapacity, the indication for a generous subtalar joint arthrodesis with planned ankle arthrodesis should always be critically examined.

## Figures and Tables

**Table 1:** Patient characteristics.

Characteristic		TTC (*n* = 216)	TT (*n* = 216)	All (*n* = 432)	*p*
Follow-up (months)	Mean	78.61	69.48	73.99	0.018
	SEM	2.73	2.70	1.93	
	Minimum	12.00	13.00	12.00	
	Maximum	154.00	152.00	154.00	
Age, years	Mean	64.02	63.67	63.84	0.760
	SEM	0.85	0.81	0.59	
	Minimum	27.00	29.00	27.00	
	Maximum	93.00	91.00	93.00	
BMI, kg/m^2^	Mean	30.18	29.63	29.91	0.361
	SEM	0.43	0.42	0.30	
	Minimum	16.40	18.60	16.40	
	Maximum	58.30	64.10	64.10	
Sex, *n* (%)	Male	135 (62.50)	134 (66.20)	278 (64.35)	0.423
	Female	81 (37.50)	73 (33.80)	154 (35.65)	
Affected side, *n* (%)	Left	111 (51.39)	95 (43.98)	206 (47.69)	0.124
	Right	105 (48.61)	121 (56.02)	226 (52.32)	
Smoker, *n* (%)	Yes	51 (23.61)	34 (15.74)	85 (19.68)	0.029
	No	159 (73.61)	181 (83.80)	340 (78.70)	
	n.a.	6 (2.78)	1 (0.46)	7 (1.62)	
Pre-existing conditions (multiple answer), *n* (%)	Associated metabolic syndrome	82 (37.96)	79 (36.57)	161 (37.27)	0.385
	Rheumatism	10 (4.63)	8 (3.70)	18 (4.17)	
	Others	44 (20.37)	29 (13.43)	73 (16.90)	
	None	45 (20.83)	57 (26.51)	102 (23.61)	
Initial injury, (multiple answer), *n* (%)	Ankle fracture	120 (55.56)	134 (62.04)	254 (58.79)	0.054
	Ankle Ligament Tear	15 (6.94)	47 (21.76)	62 (14.35)	<0.001
	Syndesmotic injury	16 (7.41)	22 (10.18)	38 (8.79)	0.196
	Failure of Total Ankle Arthroplasty	29 (13.43)	9 (4.17)	38 (8.79)	0.003
	Talar fracture	24 (11.11)	6 (2.78)	30 (6.94)	<0.001
	Others	43 (19.9)	51 (23.61)	94 (21.76)	0.251

BMI, body mass index; SEM, standard error of the mean; TTC, Tibiotalocalcaneal Arthrodesis; TT, Tibiotalar Arthrodesis.

**Table 2:** Clinical outcome with subgroups.

Measurements		TTCA (*n* = 216)	TTA (*n* = 216)	All (*n* = 432)	*p*
Olerud & Molander	Mean	43.00	58.16	50.67	<0.001
	SEM	1.68	1.67	1.24	
	Minimum	0.00	0.00	0.00	
	Maximum	100.00	100.00	100.00	
FFI-D	Mean	76.64	61.36	68.89	<0.001
	SEM	1.98	2.17	1.52	
	Minimum	15.00	5.00	5.00	
	Maximum	135.00	140.00	140.00	
SF-12 (Physical component summary)	Mean	35.52	42.49	39.07	<0.001
	SEM	0.75	0.71	0.54	
	Minimum	11.73	16.54	11.73	
	Maximum	56.63	61.22	61.22	
SF-12 (Mental component summary)	Mean	50.09	51.07	50.59	0.369
	SEM	0.81	0.72	0.54	
	Minimum	17.10	18.39	17.10	
	Maximum	68.89	71.03	71.03	
Gait after surgery as expected	As expected	80 (37.04)	83 (38.43)	163 (37.73)	0.360
	Better than expected	63 (29.17)	91 (42.13)	154 (35.65)	
	Worse than expected	63 (29.17)	40 (18.52)	103 (23.84)	
	n.a.	10 (4.63)	2 (0.93)	12 (2.78)	
Complication, Revision surgery needed (multiple answer), *n* (%)	Yes	64 (29.63)	16 (7.41)	80 (18.52)	<0.001
	No	152 (70.37)	200 (92.59)	352 (81.48)	
	Infection	23 (10.65)	5 (2.32)	28 (6.48)	<0.001
	Non-union	32 (14.81)	5 (2.32)	37 (8.57)	<0.001
Footwear (multiple answer), *n* (%)	Orthotic insoles only	41 (18.98)	72 (33.33)	113 (26.16)	0.002
	Shoe adaption	106 (49.07)	66 (30.56)	172 (39.82)	
	Others	9 (4.17)	2 (0.93)	11 (2.55)	
	Nothing special	60 (27.78)	76 (35.19)	136 (31.48)	
Return to the learned profession, *n* (%)	Yes	33 (15.28)	57 (26.39)	90 (20.83)	<0.001
Permanently unable to work, *n* (%)	Yes	49 (22.68)	26 (12.04)	75 (17.36)	<0.001
Retraining, part-time, pension, *n* (%)	Yes	134 (62.04)	133 (61.57)	267 (61.81)	>0.05

SEM, standard error of the mean; MT, metatarsal; SF-12, 12-Item Short Form Health Survey; TTCA, Tibiotalocalcaneal Arthrodesis; TTA, Tibiotalar Arthrodesis.

## Data Availability

All data intended for publication are included in the manuscript.

## References

[1] Saltzman C.L., Salamon M.L., Blanchard G.M., Huff T., Hayes A., Buckwalter J.A., Amendola A. (2005). Epidemiology of ankle arthritis: Report of a consecutive series of 639 patients from a tertiary orthopaedic center. Iowa Orthop. J..

[2] Vitiello R., Perna A., Peruzzi M., Pitocco D., Marco G. (2020). Clinical evaluation of tibiocalcaneal arthrodesis with retrograde intramedullary nail fixation in diabetic patients. Acta Orthop. Traumatol. Turc..

[3] Fischer S., Klug A., Faul P., Hoffmann R., Manegold S., Gramlich Y. (2022). Superiority of upper ankle arthrodesis over total ankle replacement in the treatment of end-stage posttraumatic ankle arthrosis. Arch. Orthop. Trauma Surg..

[4] Watts D.T., Moosa A., Elahi Z., Palmer A.J.R., Rodriguez-Merchan E.C. (2022). Comparing the Results of Total Ankle Arthroplasty vs. Tibiotalar Fusion (Ankle Arthrodesis) in Patients with Ankle Osteoarthritis since 2006 to 2020—A Systematic Review. Arch. Bone Jt. Surg..

[5] Easley M.E., Montijo H.E., Wilson J.B., Fitch R.D., Nunley J.A. (2008). Revision tibiotalar arthrodesis. J. Bone Joint Surg. Am..

[6] Cibura C., Lotzien S., Yilmaz E., Baecker H., Schildhauer T.A., Gessmann J. (2022). Simultaneous septic arthrodesis of the tibiotalar and subtalar joints with the Ilizarov external fixator-an analysis of 13 patients. Eur. J. Orthop. Surg. Traumatol..

[7] Ajis A., Tan K.J., Myerson M.S. (2013). Ankle arthrodesis vs. TTC arthrodesis: Patient outcomes, satisfaction, and return to activity. Foot Ankle Int..

[8] Cao L., Kyung M.G., Park G.Y., Hwang I.U., Kang H.W., Lee D.Y. (2022). Foot and Ankle Motion after Tibiotalocalcaneal Arthrodesis: Comparison with Tibiotalar Arthrodesis Using a Multi-Segment Foot Model. Clin. Orthop. Surg..

[9] Perisano C., Cannella A., Polichetti C., Mascio A., Comisi C., De Santis V., Caravelli S., Mosca M., Spedicato G.A., Maccauro G. (2023). Tibiotalar and Tibiotalocalcaneal Arthrodesis with Paragon28 Silverback(TM) Plating System in Patients with Severe Ankle and Hindfoot Deformity. Medicina.

[10] Deleu P.A., Piron M., Leemrijse G., Besse J.L., Cheze L., Devos Bevernage B., Lalevee M., Leemrijse T. (2022). Patients’ point of view on the long-term results of total ankle arthroplasty, tibiotalar and tibiotalocalcaneal arthrodeses. Orthop. Traumatol. Surg. Res..

[11] Lu V., Tennyson M., Zhang J., Thahir A., Zhou A., Krkovic M. (2023). Ankle fusion with tibiotalocalcaneal retrograde nail for fragility ankle fractures: Outcomes at a major trauma centre. Eur. J. Orthop. Surg. Traumatol..

[12] Gowda B.N., Kumar J.M. (2012). Outcome of ankle arthrodesis in posttraumatic arthritis. Indian J. Orthop..

[13] Maffulli N., Longo U.G., Locher J., Romeo G., Salvatore G., Denaro V. (2017). Outcome of ankle arthrodesis and ankle prosthesis: A review of the current status. Br. Med. Bull..

[14] Prissel M.A., Simpson G.A., Sutphen S.A., Hyer C.F., Berlet G.C. (2017). Ankle Arthrodesis: A Retrospective Analysis Comparing Single Column, Locked Anterior Plating to Crossed Lag Screw Technique. J. Foot Ankle Surg..

[15] Valiyev N., Demirel M., Hurmeydan O.M., Sunbuloglu E., Bozdag E., Kilicoglu O. (2021). The Effects of Different Screw Combinations on the Initial Stability of Ankle Arthrodesis. J. Am. Podiatr. Med. Assoc..

[16] Schlickewei C., Neumann J.A., Yarar-Schlickewei S., Riepenhof H., Valderrabano V., Frosch K.H., Barg A. (2022). Does Concurrent Distal Tibiofibular Joint Arthrodesis Affect the Nonunion and Complication Rates of Tibiotalar Arthrodesis?. J. Clin. Med..

[17] Gramlich Y., Neun O., Klug A., Buckup J., Stein T., Neumann A., Fischer S., Abt H.P., Hoffmann R. (2018). Total ankle replacement leads to high revision rates in post-traumatic end-stage arthrosis. Int. Orthop..

[18] Heifner J.J., Monir J.G., Reb C.W. (2021). Impact of Bone Graft on Fusion Rates in Primary Open Ankle Arthrodesis Fixated with Cannulated Screws: A Systematic Review. J. Foot Ankle Surg..

[19] Usuelli F.G., Pantalone A., Maccario C., Guelfi M., Salini V. (2017). Sports and Recreational Activities following Total Ankle Replacement. Joints.

[20] Rogero R.G., Fuchs D.J., Corr D., Shakked R.J., Raikin S.M. (2020). Ankle Arthrodesis Through a Fibular-Sparing Anterior Approach. Foot Ankle Int..

[21] Yang X.Q., Zhang Y., Wang Q., Liang J.Q., Liu L., Liang X.J., Zhao H.M. (2022). Supramalleolar Osteotomy vs. Arthrodesis for the Treatment of Takakura 3B Ankle Osteoarthritis. Foot Ankle Int..

[22] Cooper P.S. (2001). Complications of ankle and tibiotalocalcaneal arthrodesis. Clin. Orthop. Relat. Res..

[23] Crosby L.A., Yee T.C., Formanek T.S., Fitzgibbons T.C. (1996). Complications following arthroscopic ankle arthrodesis. Foot Ankle Int..

[24] Muir D.C., Amendola A., Saltzman C.L. (2002). Long-term outcome of ankle arthrodesis. Foot Ankle Clin..

